# Skeletal muscle transcriptomics identifies common pathways in nerve crush injury and ageing

**DOI:** 10.1186/s13395-021-00283-4

**Published:** 2022-01-29

**Authors:** C. A. Staunton, E. D. Owen, K. Hemmings, A. Vasilaki, A. McArdle, R. Barrett-Jolley, M. J. Jackson

**Affiliations:** MRC- Versus Arthritis Research Centre for Integrated research into Musculoskeletal Ageing (CIMA), Department of Musculoskeletal and Ageing Science, Institute of Life Course and Medical Sciences, University of Liverpool, Liverpool, L7 8TX UK

**Keywords:** Skeletal muscle, Motor neuron, RNAseq, Transcriptomic, Ageing, Crush, Neurodegeneration

## Abstract

Motor unit remodelling involving repeated denervation and re-innervation occurs throughout life. The efficiency of this process declines with age contributing to neuromuscular deficits. This study investigated differentially expressed genes (DEG) in muscle following peroneal nerve crush to model motor unit remodelling in C57BL/6 J mice. Muscle RNA was isolated at 3 days post-crush, RNA libraries were generated using poly-A selection, sequenced and analysed using gene ontology and pathway tools. Three hundred thirty-four DEG were found in quiescent muscle from (26mnth) old compared with (4-6mnth) adult mice and these same DEG were present in muscle from adult mice following nerve crush. Peroneal crush induced 7133 DEG in muscles of adult and 699 DEG in muscles from old mice, although only one DEG (*ZCCHC17*) was found when directly comparing nerve-crushed muscles from old and adult mice. This analysis revealed key differences in muscle responses which may underlie the diminished ability of old mice to repair following nerve injury.

**Supplementary Information:**

The online version contains supplementary material available at 10.1186/s13395-021-00283-4.

## Introduction

Loss of skeletal muscle mass and function occurs over a substantial portion of later life and plays a crucial role in the development of frailty, leading to increased risk of falls, immobility, loss of independence and declining quality of life [68, 93, 112]. The loss of muscle mass and function with age is primarily due to the loss of muscle fibres and an atrophy and weakening of remaining fibres in humans and rodents [8, 62, 63, 85]. The maximum isometric force decreases to a greater extent than muscle mass during ageing in mice in a similar manner to humans, even when expressed relative to the cross-sectional area of the muscle [30, 38]. Thus, the age-related loss of muscle strength cannot be solely explained by the loss of muscle mass: both muscle “quantity” and “quality” decline in old animals [25], with evidence for some change to a slower fibre type which is particularly apparent in human subjects [63].

The mechanisms responsible for the loss of muscle mass and function have not been fully elucidated, but it has been estimated from electromyography studies that there are 40% fewer motor units by age 70 [10, 84]. In young and adult humans and animals, motor unit turnover occurs during everyday activities and is repaired by sprouting and regrowth of axons from the damaged nerve leading to rapid re-innervation of the muscle at the neuromuscular junctions (NMJs) [24]. This complex process involves coordinated responses by the damaged axon, terminal Schwann cells and denervated muscle fibre [52, 60]. With increasing age, it is proposed that re-innervation does not occur appropriately and is less efficient and re-innervation occurs by sprouting from adjacent axons rather than the originally damaged axon [60]. This process leads to formation of “giant” motor units which are eventually lost [24, 71]. Denervation can lead to atrophy and loss of muscle fibres [65, 76]. Studies of the innervation of individual muscle fibres in old mice show NMJs with a variety of age-related structural alterations, including axonal swellings, sprouting, synaptic detachment, partial or complete withdrawal of axons from some postsynaptic sites, and fragmentation of the postsynaptic specialisation [11, 105]. It is unclear whether the changes in NMJs are initiated by changes in the motor neuron and/or the muscle fibre. Our data showed that in muscles of old mice, ∼15% of muscle fibres are completely denervated and ∼80% of NMJs disrupted [108] and studies using a mouse model of accelerated muscle ageing (mice lacking CuZn superoxide dismutase; SOD1) indicate that the integrity of motor neurons and the NMJ dictate whether muscle fibres undergo accelerated ageing [23, 90–92, 109, 114].

The process of axonal regeneration and regrowth is complex, but elegant studies in tractable models such *Caenorhabditis elegans* and Zebrafish have identified key processes and signalling pathways involved in regrowth following damage [17, 42, 97]. Axonal sprouting can be induced by nerve transection, nerve crush or paralysis of muscle [100] and these processes lead to release of “sprouting factors” early after injury (as early as 3 days) from the target muscles, Schwann cells and infiltrating cells [98]. These factors are believed to cause “self-repair” and facilitate re-growth of the damaged axons and aid recruitment of the re-growing axon to the denervated muscle fibre. This re-growth is evident from intra-vital imaging of the NMJ in adult mice following nerve crush [99], but the response in aged mice and the mechanisms behind the responses are yet to be defined. Data indicate that lack of neuromuscular transmission is the prime stimulus for release of sprouting factors and these factors are anterograde signals, from Schwann cells or muscle fibres as early as 3 days post injury that ultimately leads to sprouting [28, 98]. Previous microarray studies in skeletal muscle have highlighted how ageing resulted in a marked stress response and a lower expression of metabolic and biosynthetic genes [111]; studies exploring the mechanisms behind weightlessness-induced skeletal muscle atrophy again highlighted the activation of stress responses, activation of proteolytic systems and, to a certain degree, inflammatory responses in adult rodents; however, the response to nerve injury/crush in terms of differential gene expression (DEG) in adult and old subjects has never been reported.

The present study examined the transcriptomic profile of anterior tibialis (AT) muscles from adult and old mice, as well as from a subset of adult and old mice that had undergone a crush injury to the peroneal nerve. We aimed to identify pathways underlying age-related decline in skeletal muscle and differences between the responses of muscle from adult and older mice to nerve crush.

## Methods

### Experimental animals

Adult (6–8 months) and old (26 months) C57BL/6 J male mice were obtained from Charles River (North Carolina, USA). All animals were housed in a temperature-controlled room (22–25 °C) with food and water provided ad libitum on a 12-h light/dark cycle in the University of Liverpool animal facilities. All experimental procedures were performed under a UK Home Office licence (Home Office licence number P391895CA, approved 15/06/17) and complied with the UK Animals (Scientific Procedures) Act 1986 and received ethical approval from the University of Liverpool AWERB (Ethical approval number AWC0066, approved 23/3/17).

### Surgical peroneal nerve crush procedure

Mice were anaesthetised using isoflurane, the hind limb was shaved, antiseptic skin cleanser (Videne) applied and buprenorphine given (100 μl, 0.3 mg/ml). All animals were maintained under gaseous anaesthesia throughout and all surgery carried out as per Staunton et al. [99]. A small incision (5 mm) was made on the outer side of the limb and the peroneal nerve exposed. The nerve was crushed using a curved micro needle holder for 10 s, and complete nerve crush was confirmed by the appearance of a translucent band across the nerve [101]. The surrounding connective tissue was then placed back over the nerve and skin sutured using Clinisorb 6.0 sutures (Akacia Medical, UK) and the mice allowed to recover in a heated chamber with moist food available until normal movement, feeding and exploratory behaviour were observed. Groups of mice were allowed to recover for 3 days before being sacrificed when tissues were dissected and used for further analyses. A total of 20 mice were used for this study; *n* = 5/adult control, *n* = 5/ adult crush, *n* = 5/old control, *n* = 5/old crush.

### RNA isolation and library preparation for RNAseq

Mice were killed by a Schedule 1 procedure (cervical dislocation) and the AT muscle dissected and frozen in liquid nitrogen. RNA extraction was performed using the RNeasy Fibrous Tissues kit (Qiagen, UK) as per the manufacturer’s instructions. Approx. 30 mg of tissue was lysed and homogenised using an ultra turrax (IKA Homogeniser, Sigma, UK) in buffer RLT before proteinase K digestion steps, total RNA bound followed by several washing steps and DNase digestion and total RNA eluted. Total RNA integrity was confirmed using the Bioanalyzer (Agilent Technologies, USA). Ribosomal RNA (rRNA) was depleted from RNA samples by using the Ribo-Zero™ rRNA Removal Kit (Epicentre, USA) in accordance with the instructions of the manufacturer.

### RNA data processing

DNA free total RNA was selected for poly A using NEB Next® Poly(A) mRNA Magnetic isolation Module. RNA–Seq libraries were prepared from the enriched material using the NEBNext®Ultra™Directional RNA Library Prep Kit for Illumina®#E7420. Libraries were purified using AMPure XP beads. Each library was quantified using Qubit and the size distribution assessed using the Agilent 2100 Bioanalyser. The quantity was assessed using a Qubit® dsDNA HS Assay Kit, while the quality and average size was assessed using the High Sensitivity DNA Kit. Subsequently, qPCR assay, designed to specifically detect adapter sequences flanking the Illumina libraries, was performed using an Illumina® KAPA Library Quantification Kit (Kapa Biosystems, Wilmington, USA). The RNA libraries were sequenced on an Illumina® HiSeq 4000 platform with version 1 chemistry using sequencing by synthesis (SBS) technology to generate 2 × 150 bp paired-end reads. Sequencing was performed at the Centre for Genomic Research, University of Liverpool (https://www.liverpool.ac.uk/genomic-research/).

### Bioinformatic analysis and read alignment

Base-scaling and de-multiplexing of indexed reads was performed by CASAVA version 1.8.2 (Illumina) to produce FASTQ format. The raw FASTQ files were trimmed to remove Illumina adapter sequences using Cutadapt version 1.2.1 [70]. The reads were further trimmed to remove low-quality bases, using Sickle version 1.200 with a minimum window quality score of 20. After trimming, reads shorter than 20 bp were removed. Reads were then aligned to the genome sequences using TopHat version 2.1.0 [59] and transcript assembly conducted using HTSeq2.

### Differential gene expression analysis

Raw counts were subsequently used as the input into R (version 1.2.5042) to utilise the DESeq2 package [67] for identifying differentially expressed genes (DEG). Data was assessed using pairwise comparisons, correlation heatmaps and PCA plots generated from normalised count data using R. The R packages “factorMineR” and “factoextra” were implemented for PCA analysis and the “heatmap.2” function from “gplots” was used to plot heatmaps [61].

Fold changes and the threshold of false discovery rate (FDR) adjusted *p* values < 0.05, generated using the Benjamini-Hochberg approach and in some cases a 1.4 log2 fold change (Log2FC) from the DEG, were used to perform functional analysis, specifically enrichment analysis with Gene Ontology (GO) Biological Process using the Panther Classification System [74], Kyoto Encyclopedia of Genes and Genomes (KEGG) pathway enrichment analysis using the “enrichKEGG” command from clusterProfiler v. 3.8.1 [113] from Bioconductor v. 3.7 [36] with mouse as the reference organism. The GO terms and KEGG pathways with FDR < 0.05 were considered to be significantly enriched.

Further bioinformatics to determine the interactions of age and/or crush-related DEG, canonical pathway, upstream regulator, causal/master regulators and network analyses from these were performed using Ingenuity Pathway Analysis package (IPA) (IPA, Qiagen Redwood City, USA) “Core Analysis” settings. The core analysis was carried out with the settings of indirect and direct relationships between molecules based on experimentally observed data, and data sources were considered in mouse databases in the Ingenuity Knowledge Base. All network scores were calculated by IPA as negative exponent of the *p* value calculated for that network as described by Calvano et al. [9]. In addition, predicted protein-protein interactions for the list of differentially expressed proteins and the resulting network were retrieved and constructed using the STRING database version 9.0 (http://string-db.org). For the adult control vs old control cohort, an additional analysis was performed using the SarcoAtlas database to compare results here to the mRNA profiles of the TA skeletal muscles of male C57BL/6JRj mice from adult (10 month) and old (30 months) (https://sarcoatlas.scicore.unibas.ch/) [6, 40, 41]. This comparison used only wild-type mice and not those that had any drug treatment or specialised diet. Those genes deemed to have DE expression were then utilised if the FDR < 0.05.

We measured cluster separation as Euclidean distance between treatment groups and tested for statistical significance with the R ClusterSignificance package [94]. Equivalence testing was conducted using R based around the practices of Hilden [43] and Lakens [57], using normalised count data and the packages “EQUIVNONINF” and “TOSTER”.

Full data are available on the EBI array express database with accession number: E-MTAB-10601.

## Results

**Fig. 1:**
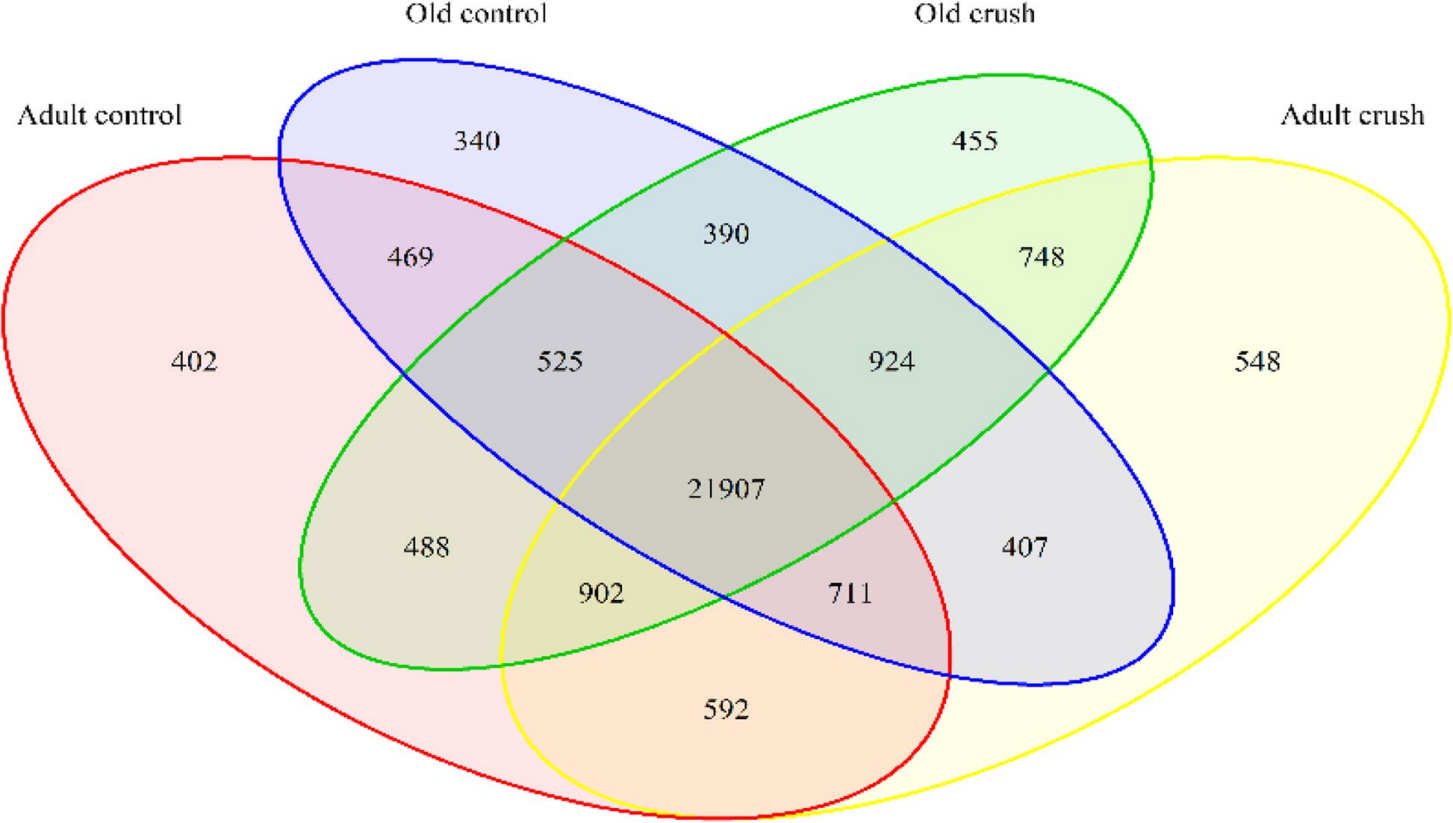
Total genes detected (not differentially expressed) for each contrast

RNAseq analysis was conducted on AT muscle from control adult and old male mice as well as mice that had undergone a peroneal nerve crush model of denervation and regeneration. The AT muscle was used for all studies, 2 × 150 bp paired-end reads were generated, then data processed, and a total of 29,842 different gene transcripts were detected across 20 samples. The Venn diagram in Fig. 1 shows all genes detected in each treatment group and where overlap occurred. A total of 21,907 genes were commonly transcribed in all treatment groups.

**Fig. 2:**
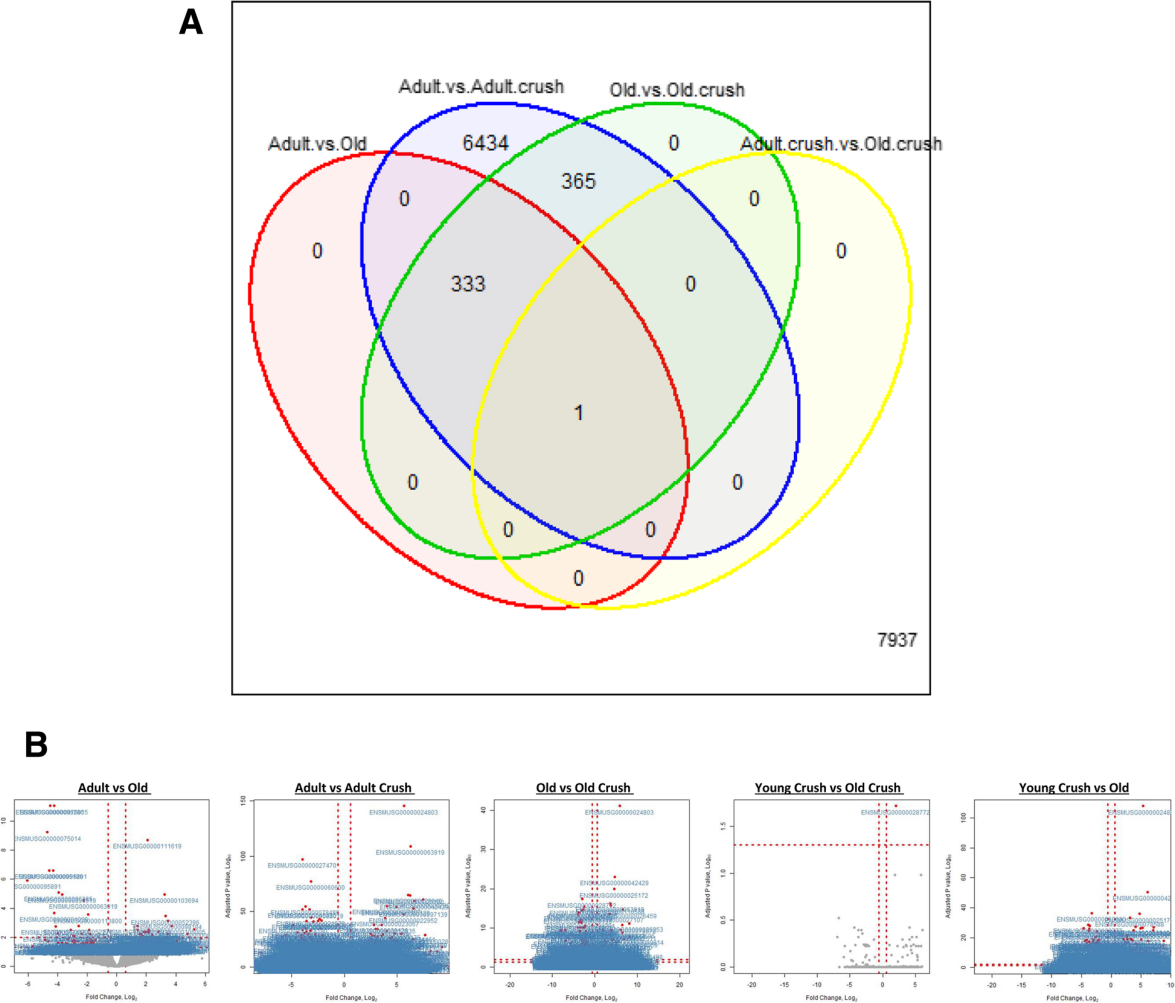
Comparisons between the differentially expressed genes for skeletal muscle from adult (6 months) and old (26 months) mice at rest and 3 days post peroneal nerve crush. **A** Venn diagram shows the overlap of the DEG between all contrasts (FDR < 0.05, *n* = 5/group). A total of 334 genes were DE between control tissue from adult and old mice, 7133 DE between adult control and crushed, 699 DE between old control and old crushed, 1 DE gene from adult crush vs old crushed and 1745 DEG revealed when examining the adult crush vs old control. **B** Volcano plots show the FDR for difference in expression in skeletal muscle genes between all contrasts for each gene detected plotted against the log2 fold-change. Genes with an FDR < 0.05 are depicted in red with the ENSMBL ID associated also labelled; those non-significant genes are outlined as grey dots

Differential expressional analysis was conducted using the DESEQ2 tool [67], and Table 1 outlines the number of differentially expressed genes (DEG), including both those up- and down-regulated across each different comparison. The Venn diagram in Fig. 2A details where there was overlap in DEG between comparisons. The largest number of DEG was found by comparison of adult vs adult crush cohort where a total of 7133 DEG were detected, with a similar number of genes increased and decreased (FDR < 0.05, *n* = 5/cohort) (Table 1). The data for each contrast can also be visualised in the volcano plots in Fig. 2B showing individual genes, fold changes and level of significance.

**Table 1:** Comparison of muscles from adult and old mice: DESEQ2 analysis revealed differentially expressed genes

	Adult control vs Old control	Adult vs Adult crush	Old vs Old crush	Adult crush vs Old crush	Adult crush vs Old control
Total DEG (padj< 0.05)	334	7133	699	1	1745
Number DEG upregulated	199	3656	292	1	852
Number DEG downregulated	135	3477	407	0	893

### Adult control vs old control—which genes and pathways are differentially expressed in skeletal muscle from old compared with adult mice?

A total of 334 DEG were found from a comparison of muscle from adult and old control mice; 199 of which were upregulated and 135 downregulated in muscles of old compared with adult mice (FDR > 0.05, Table 1). This included changes in genes anticipated such as a downregulation of REDOX-related glutathione-S-transferase (*Gstp2*), as well as downregulation of the complex 1-related gene NADH:ubiquinone oxidoreductase (*Ndufa13*), and upregulation of heat shock proteins (e.g. *hsp8*). The 5 significantly DEG that were most upregulated (in terms of Log2FC) were Leucine-rich repeat kinase 2/PARK8 (*Lrrk2*), Complement Factor H (*Cfh*), ATPase Na+/K+ Transporting Subunit Alpha 4 (*Atp1a4*), Kelch Repeat and BTB Domain Containing 7 (*Kbtbd7*) and Vitamin K-Dependent Protein S (*Pros1*). The 5 significantly DEG that were most downregulated (in terms of Log2FC) were Syntaxin 11 (Stx11), WD repeat and HMG-box DNA binding protein 1/CTF4 (*Wdhd1*), Angiopoietin-like protein 3 (*Angptl3*), TOX High Mobility Group Box Family Member 2 (Tox2) and Galactose-3-O-Sulfotransferase 3 (*Gal3st3*).

**Fig. 3:**
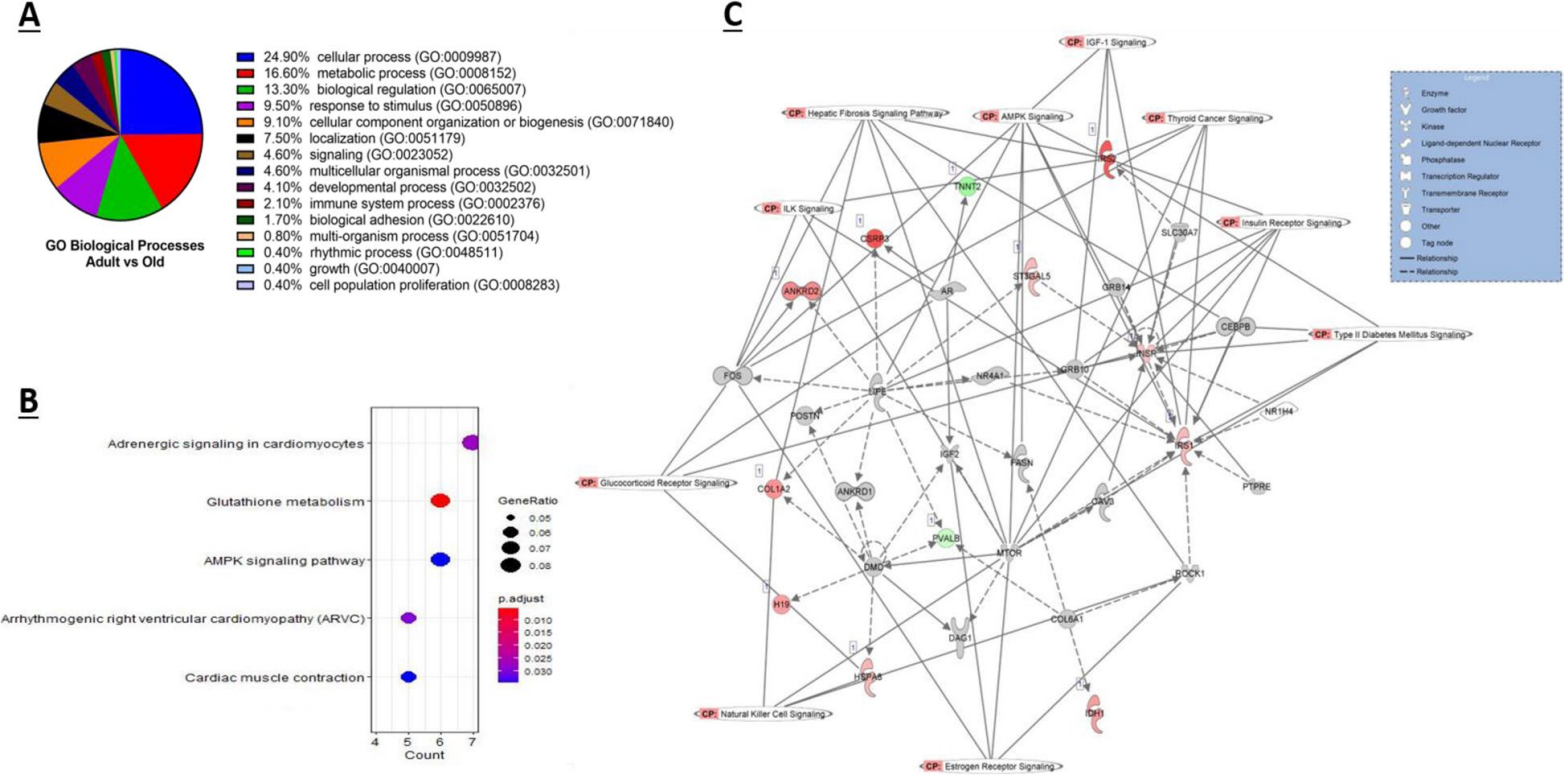
DEG between adult and old murine skeletal muscle. **A** Enriched GO biological process terms of age-associated DEG with a *p*-value < 0.05 are listed and outlined in the pie chart. **B** Top 5 KEGG enriched pathways of DEG with the associated gene ratio and BH adjusted *p* value (FDR) colour chart produced using enrichKEGG. **C** Top scoring networks derived from the DE genes identified for contrast 1 with the 10 associated significantly enriched pathways for the network also identified on the figure with CP tag. Green nodes, increased expression in old; red nodes, lower expression in old; white nodes, genes not differentially expressed with age. Intensity of colour is related to higher fold-change

Gene ontology (GO) analysis was conducted using PANTHER and when examining the DEG obtained from adult vs old controls, a total of 15 shared GO biological processes were reported, with approximately 25% of the DEG classified into the cellular process category (GO:0009987), which includes cell growth and/or cell maintenance processes (Fig. 3A). Statistical overrepresentation tests revealed both glutathione peroxidase activity (GO:0004602) and protein binding (GO:0005515) significantly overrepresented in terms of GO molecular functions (*p* < 0.05, Fisher exact test). The TRAF2-GSTP1 complex (GO:0097057) was the most significantly overrepresented GO term for cellular components, followed by endoplasmic reticulum exit site (GO:0070971), I band (GO:0031674) and Z disc (GO:0030018).

KEGG pathway enrichment analysis was performed using enrichKEGG; a total of 5 significantly enriched KEGG categories were uncovered, as shown in Fig. 3B. Of particular interest, glutathione metabolism and AMPK signalling pathways which have both been extensively studied in skeletal muscle were significantly enriched based on DEG from adult and old skeletal muscle (Fig. 3B, FDR < 0.05).

Using the well-established Ingenuity Pathway Analysis (IPA) (Qiagen Redwood City, USA), the DEG from a comparison of muscle from adult vs old were further analysed based on an FDR of < 0.05 with a 1.4 log2 fold change cut off/threshold (Log2FC). A total of 16 significantly enriched canonical pathways were identified (when scored using Fisher’s Exact Test) as well as 17 upstream regulators and 7 causal networks (summarised outputs in Table 2). The top 10 enriched canonical pathways with a log *p* value > 1.3 as well as the representative DEG in each canonical pathway are listed in Table 3. When the enriched KEGG pathways (Fig. 3B) were compared to those top canonical pathways extracted from the Ingenuity knowledge base (Table 2), AMPK signalling was significantly enriched by both KEGG and IPA.

**Table 2:** Comparison of muscles from adult and old mice: summary of IPA results for all contrasts

	Canonical Pathways	Upstream Regulators	Causal networks/master regulators
Adult control vs old control	16/167	17	7
Adult control vs adult crush	41/516	17	8
Old control vs old crush	22/278	49	10
Adult crush vs old crush	n/a	n/a	n/a
Adult crush vs old control	55/509	35	15

**Table 3:** Comparison of muscles from old and adult mice: the top 10 canonical pathways identified from the IPA knowledge database that involve DE (adjusted *P* < 0.05 and 1.4 log2 fold change) protein coding genes

Ingenuity Canonical Pathways	-log(*p*-value)	Ratio
Insulin Receptor Signalling	2.48	0.06
AMPK Signalling	2.44	0.05
Geranylgeranyldiphosphate Biosynthesis	1.95	1.00
Dopamine Receptor Signalling	1.87	0.07
IL-9 Signalling	1.86	0.13
Apelin Liver Signalling Pathway	1.81	0.12
Th1 Pathway	1.72	0.06
Type II Diabetes Mellitus Signalling	1.66	0.04
Trans, trans-farnesyl Diphosphate Biosynthesis	1.65	0.50
Role of JAK2 in Hormone-like Cytokine Signalling	1.63	0.10

The advanced analytics of IPA allow identification of upstream regulators that may be causal regulators of observed gene changes (“master regulators”). Using this method, the 5 most significant upstream regulators were dystrophin (DMD), solute carrier family 30 member 7 (*SLC30A7*), lipase E (*LIPE*), androgen receptor (*AR*) and collagen type VI alpha 1 chain (*COL6A1*). Four master regulators were identified for the Adult vs Old DEG expression data: TAR DNA binding protein (*TARDBP*), growth factor receptor bound protein 14 (*GRB14*), growth factor receptor bound protein 10 (*GRB10*) and CCAAT enhancer binding protein beta (*CEBPB*).

Six key networks of interconnected molecules were identified in our data by IPA network analysis algorithms. They consist of several focus molecules and are constructed for connectivity and all network scores were calculated by IPA as negative exponent of the *p* value calculated for that network as described by [9]. The highest scoring network had a score of 13 and comprised of 12 focus molecules and is shown in Fig. 3C. The network shown is also overlaid with 10 canonical pathways that are associated with the DEG in the network. The main biological functions that this network is associated with were carbohydrate metabolism, molecular transport and small molecule biochemistry. The network comprised of several genes including those encoding heat shock proteins, dystrophin, FOS and collagen. All interactions/relationships between those DEG are linked by different lines and legend are also shown.

Using the interactive SarcoAtlas database, we compared the DEG here against a mRNA profile of the *Tibialis anterior* skeletal muscles of 10- and 30-month-old wild-type mice. Using the gene expression output with an FDR threshold of < 0.05, a total of 1696 gene expression profiles were outputted; this was then compared to the 334 DEG found in this study and a total of 28 shared genes were uncovered. The main difference between RNA-Seq and microarrays is that the former allows for full sequencing of the whole transcriptome while the latter only profiles predefined transcripts/genes through hybridization so it was not surprising that the overlap was only 28 genes. These results are presented in supplementary Table S1.

Equivalence testing was then utilised to explore the potential similarity between the two populations; this examined all the genes detected to assess the number of genes that were statistically similar in expression between the adult control and old control populations. This non-parametric test used normalised gene counts and identified 2225/21388 genes that were statistically similar in expression between the adult and the old control samples.

### Adult control vs adult crush—which genes and pathways are differentially modified in skeletal muscle by crush damage to the peroneal nerve of adult mice?

In order to understand why motor unit turnover differs with age and how different ages respond to injury, a peroneal nerve crush model was undertaken and the AT muscle from both control and crushed tissue was examined 3 days post nerve crush. RNAseq analysis detected a total of 29,842 genes, with 23% of those genes differentially expressed between adult control and crushed tissue (7133 DEG, *n* = 5 per group, FDR < 0.05, Table 1). A total of 3656 genes were upregulated and 3477 downregulated following crush. The 5 significantly DEG genes that were most upregulated (in terms of Log2FC) were Cation Channel Sperm Associated 1 (*Catsper1*), Growth/differentiation factor 5/ BMP 14 (*Gdf5*), Neuronal acetylcholine receptor subunit alpha-9 (*Chrna9*), FAT Atypical Cadherin 2 (*Fat2*) and Cholinergic Receptor Nicotinic Gamma (*Chrng*) and the 5 most significantly downregulated genes were Synaptotagmin 8 (*Syt8*), Aquaporin 4 (*Aqp4*), Kelch Repeat And BTB Domain Containing 13 (*Kbtbd13*), Methyltransferase Like 21C (*Mettl21c*) and Monoacylglycerol O-Acyltransferase 2 (*Mogat2*).

**Fig. 4:**
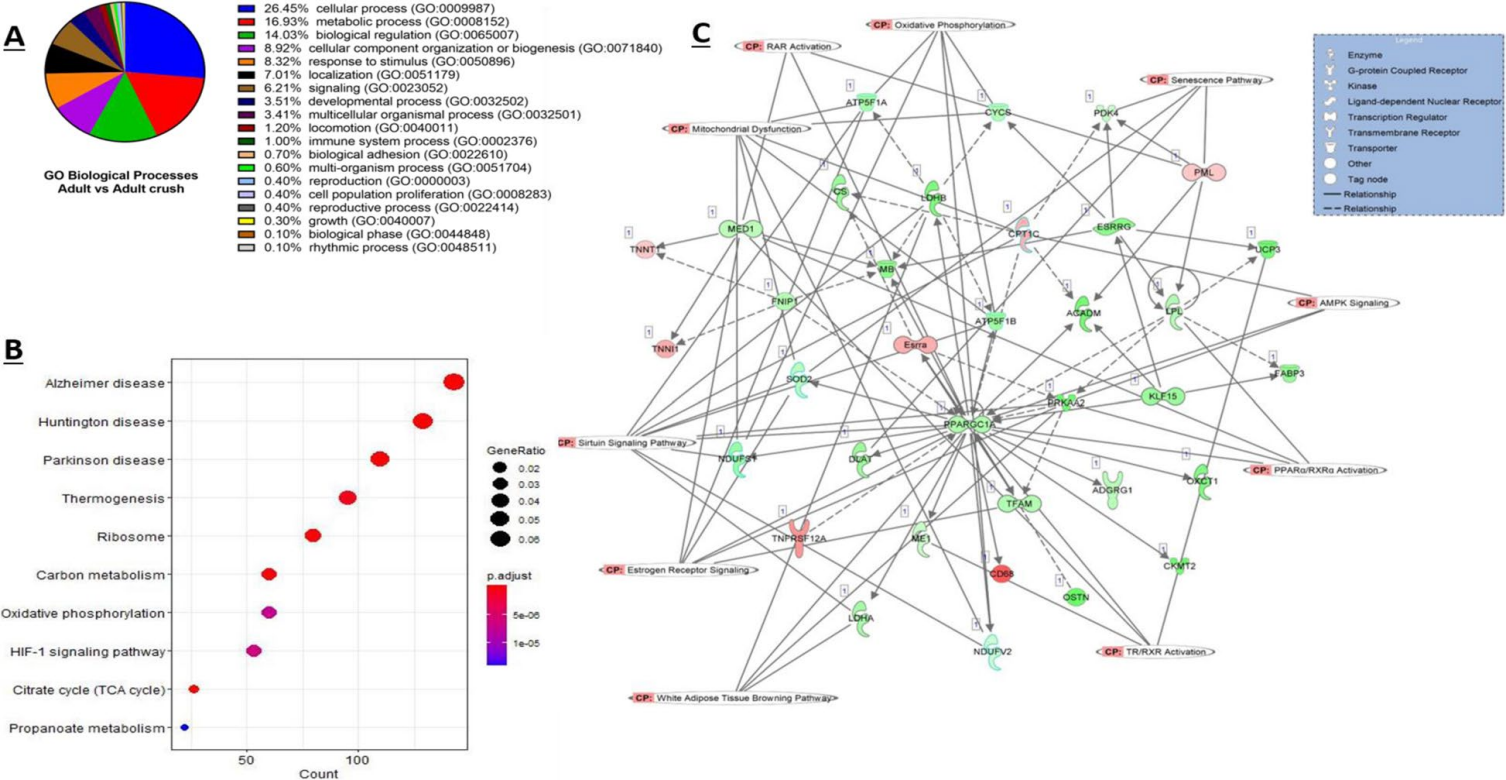
DEG between muscle from adult mice pre and post nerve crush. **A** Enriched GO biological process terms of crush associated DEG with a *p*-value < 0.05 are listed and outlined in the pie chart. **B** Top 5 KEGG enriched pathways of DEG with the associated gene ratio and BH adjusted *p* value (FDR) colour chart produced using enrichKEGG. **C** Top scoring networks derived from the DE genes identified for the comparisons between control and crush with the 10 associated significantly enriched pathways for the network also identified on the figure with CP tag. Green nodes, increased expression in old; red nodes, lower expression in old; white nodes, genes not differentially expressed with age. Intensity of colour is related to higher fold-change

GO analysis with PANTHER on the up- and down-regulated DEG from adult control and adult crush samples revealed 21 enriched biological pathways. The top three enriched processes and the percentage of genes classified to these categories being cellular process (26.4%, GO: 0009987), metabolic process (16.9%, GO: 0008152) and biological regulation (14.0%, GO: 0065007) (Fig. 4A). The three most significant biological processes using the statistical overrepresentation tests were the 2-oxoglutarate metabolic process (GO:0006103) and the tricarboxylic acid cycle (GO:0006099) and acyl-CoA biosynthetic process (GO:0071616) (*p* < 0.05, Fisher exact test). The three most significantly overrepresented GO molecular functions were structural constituent of ribosome (GO:0003735), rRNA binding (GO:0019843) and electron transfer activity (GO:0009055) (*p* < 0.05, Fisher exact test).

Further analysis with KEGG revealed a total of 77 KEGG pathways significantly enriched with adult control versus adult crush DEG (FDR < 0.05). Figure 4B shows the 10 most significantly enriched pathways. Several neurodegenerative disease pathways were significantly enriched (Huntington disease (mmu05016), Parkinson disease (mmu05012) and Alzheimer disease (mmu05010)), as well ageing-associated pathways such as FoxO signalling (mmu04068), HIF-1 signalling pathway (mmu04066), PI3K-Akt signalling (mmu04151) and oxidative phosphorylation (mmu00190) (Fig. 4B).

Ingenuity pathway analysis was conducted to further investigate the mechanisms behind the changes seen with nerve crush and reveal key canonical pathways, upstream regulators and causal networks. A total of 41 out of 516 detected canonical pathways were significantly enriched (*p* < 0.05, Fisher’s exact test, Table 4), as well as 17 upstream regulators and 8 causal networks/master regulators uncovered.

**Table 4:** Comparison of muscle from control adult mice and adult mice following nerve crush: the top 10 canonical pathways from the IPA knowledge database that involve DE (adjusted *P* < 0.05 and 1.4 log2 fold change) protein coding genes

Ingenuity Canonical Pathways	-log(*p*-value)	Ratio
TCA Cycle II (Eukaryotic)	8.62	1
EIF2 Signalling	3.63	0.44
mTOR Signalling	3.13	0.42
Oestrogen Receptor Signalling	2.54	0.38
Sirtuin Signalling Pathway	2.34	0.38
Production of Nitric Oxide and Reactive Oxygen Species in Macrophages	2.26	0.41
D-myo-inositol-5-phosphate Metabolism	2.08	0.42
3-phosphoinositide Degradation	2.05	0.41
D-myo-inositol (1,4,5,6)-Tetrakisphosphate Biosynthesis	2.01	0.42
D-myo-inositol (3,4,5,6)-tetrakisphosphate Biosynthesis	2.01	0.42

Several canonical pathways including those linked to protein homeostasis, integrin signalling, interleukin signalling, mitochondrial dysfunction and senescence were identified and the 10 most enriched pathways and the molecules associated to the specific pathways and respective log fold change values are presented in Table 4. The most enriched canonical pathway was the TCA cycle II (consisted of 16 molecules and log *p* value 8.62). A total 17 upstream regulators were significantly associated with the DEG from adult control compared to adult crush and included transcriptional regulators together with receptors and cytokines, the 5 most significant upstream regulators were: lipase E (*LIPE*), Oestrogen-Related Receptor Gamma (*ESRRG*), PPARG coactivator 1 alpha (*PPARGC1A*), Uncoupling protein 1 (also termed Thermogenin; *UCP1*) and Smoothelin Like 1 (*SMTNL1*). Master regulators identified included a variety of molecular types such as transcriptional regulators, transports and enzymes and the top 5 master regulators were Oestrogen-Related Receptor Gamma (*ESRRG*), Histone deacetylase 4 (*HDAC4*), Uncoupling protein 1/ Thermogenin (*UCP1*), Smoothelin Like 1 (*SMTNL1*) and Alpha-1-syntrophin (*SNTA1*).

IPA analysis identified 25 significant networks in total, 3 of which were all associated with 35 focus molecules and had a score of 13. The top network is shown in Fig. 4C where the canonical pathways associated with the network were overlaid. This network comprised of several genes including superoxide dismutase (*SOD2*), the pan-macrophage marker CD68, Malic Enzyme 1 (often termed NADP-Dependent Malic Enzyme (*ME1*)) and Mitochondrial creatine kinase (*MtCK*), and the most enriched biological functions this network was associated with were energy production, molecular transport and nucleic acid metabolism.

### Old control vs old crush—which genes are differentially expressed following crush injury to the peroneal nerve in old mice?

In order to understand how muscle from old mice responds to motor nerve crush injury, the AT muscle from 26-month-old mice was compared to 26-month-old muscle that had been subjected to peroneal nerve crush 3 days prior. The transcriptomic analysis revealed a total of 699 genes were differentially expressed out of the 29,842 that were detected (*n* = 5 per group, FDR < 0.05, Table 1). Out of these 699 DEG, a total of 292 were significantly upregulated and 407 significantly downregulated.

The 5 most significantly upregulated DEG (in terms of LogFC) were Cholinergic Receptor Nicotinic Gamma Subunit (*Chrng*), Cation Channel Sperm Associated 1 (*Catsper*), Tumour Protein P53 Pathway Corepressor 1 (*Trp53cor1*), Lectin Mannose Binding 1 (Lman1l /ERGIC-53) and Chitinase 3-like-1 (*Chil1*). The 5 most downregulated genes (in terms of LogFC) were Fructosamine 3 Kinase (*Fn3k*), EMAP Like 6 (*Eml6*), Outer dense fibre protein 3-like protein 2 (*Odf3l2*), Potassium Voltage-Gated Channel Modifier Subfamily G Member 4 (*Kcng4*) and Leucine Rich Repeat Containing 38 (*Lrrc38*).

**Fig. 5:**
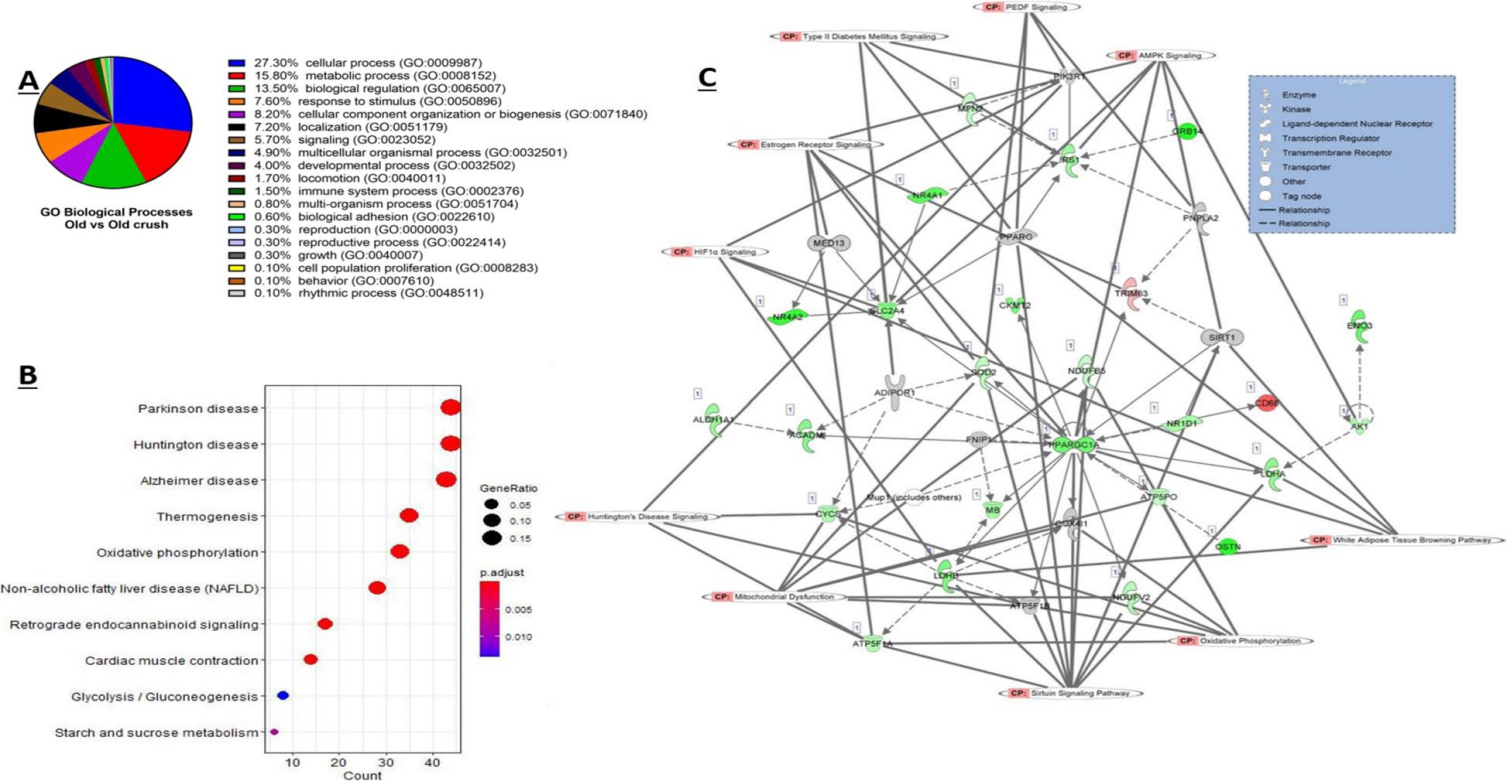
DEG between muscles from old mice pre and post nerve crush. **A** Enriched GO biological process terms associated with DEG with a *p*-value < 0.05 are listed and outlined in the pie chart. **B** Top 5 KEGG enriched pathways of DEG with the associated gene ratio and BH adjusted *p* value (FDR) colour chart produced using enrichKEGG. **C** Top scoring networks derived from the DE genes identified for the comparisons between old control and crush with the 10 associated significantly enriched pathways for the network also identified on the figure with CP tag. Green nodes, increased expression in old; red nodes, lower expression in old; white nodes, genes not differentially expressed with age. Intensity of colour is related to higher fold-change

Gene ontology analysis of old control versus old crush DEG detected 19 associated biological processes (Fig. 5A). The highest percentage of DEG were classified to cellular process (GO: 0009987), metabolic process (GO: 0008152) and biological regulation (GO: 0065007), 27.9%, 15.8% and 13.5% respectively; these results were consistent with the comparisons of muscles from adult vs old control mice and adult control vs adult nerve crushed mice discussed above. In terms of PANTHER pathways, inflammation mediated by chemokine and cytokine signalling pathway (P00031) was the highest scoring pathway with 8.7% of genes from the old vs old crushed data set classified to this. When examining the statistically overrepresented GO functions, glycogen catabolic process (GO:0005980), glucan catabolic process (GO:0009251), mitochondrial electron transport, NADH to ubiquinone (GO:0006120), and mitochondrial electron transport, ubiquinol to cytochrome c (GO:0006122) were the most significant GO biological processes (*p* < 0.05, Fisher exact test). The statistically overrepresented cellular components were mitochondrial proton-transporting ATP synthase complex, coupling factor F(o) (GO:0000276), proton-transporting ATP synthase complex, coupling factor F(o) (GO:0045263) and mitochondrial proton-transporting ATP synthase complex (GO:0005753) (*p* < 0.05, Fisher exact test). In terms of GO molecular functions, the 3 most significantly overrepresented functions were pyrimidine nucleotide binding (GO:0019103), proton-transporting ATP synthase activity, rotational mechanism (GO:0046933) and NADH dehydrogenase activity (GO:0003954) (*p* < 0.05, Fisher exact test).

KEGG enrichment analysis revealed that 12 out of a total of 259 detected KEGG pathways were significantly enriched in the old and old crush DEG dataset (FDR < 0.05), the 10 most enriched pathways are shown in Fig. 5B below. Adult crush genes that are associated with three neurodegenerative disease pathways were enriched: Parkinson disease (mmu05012), Alzheimer disease (mmu05010) and Huntington disease (mmu05016). Other pathways also enriched in the old crush DEG included oxidative phosphorylation (mmu00190) as well as calcium signalling (mmu04020) and thermogenesis (mmu04714).

Twenty-two significantly canonical pathways enriched pathways were identified in the DEG obtained comparing old control vs old crushed tissue were those with a *p* value < 0.05. These include oxidative phosphorylation, mitochondrial dysfunction and sirtuin signalling which were the top 3 enriched pathways. The top 10 canonical pathways are shown in Table 5.

**Table 5:** Comparison of muscle from control old mice and old mice following nerve crush: the top 10 canonical pathways from the IPA knowledge database that involve DE (adjusted *P* < 0.05 and 1.4 log2 fold change) protein coding genes

Ingenuity Canonical Pathways	-log(p-value)	Ratio
Oxidative Phosphorylation	17.1	0.33
Mitochondrial Dysfunction	13.8	0.23
Sirtuin Signalling Pathway	7.56	0.14
Glycolysis I	3.46	0.31
Aryl Hydrocarbon Receptor Signalling	3.31	0.13
Glycogen Degradation II	3.19	0.6
Calcium Signalling	3.13	0.11
Xenobiotic Metabolism PXR Signalling Pathway	2.92	0.12
Glycogen Degradation III	2.9	0.5
Pyruvate Fermentation to Lactate	2.77	1

Utilising the causal network analysis feature as part of the IPA knowledge base, we were able to detect and report here novel master upstream regulators that may be governing the differentially expressed genes in the old control vs old crush data set. A total of 49 upstream regulators which included transcriptional regulators, enzymes, ligand-dependent receptors, cytokines and transporters were detected and significantly overlapped (*P* < 0.05). The 5 most significantly enriched upstream regulators were peroxisome proliferator-activated receptor gamma coactivator 1-alpha (*PGC-1α*) (*PPARGC1A*), smoothelin-like protein 1 (*SMTNL1* often termed *CHASM*), mammalian target of rapamycin (*MTOR*), adiponectin receptor 1 (*ADIPOR1*) and prospero homeobox protein 1 (*PROX1*) (Fig. 5C).

Ten master regulators were identified in our dataset, that is, regulators that may not be listed directly in our dataset but are predicted to be key controllers that may activate or inhibit other pathways. The top 5 master regulators detected were smoothelin-like protein 1 (*SMTNL1*), peroxisome proliferator-activated receptor gamma coactivator 1-alpha (*PGC-1α*) (*PPARGC1A*), prospero homeobox protein 1 (*PROX1*), collagen alpha-1(VI) (*COL6A1*) and histone deacetylase 4 (*HDAC4*).

When comparing the aged control muscle to that that had been subjected to a nerve crush, a total of 15 significant networks were detected that could have a key biological role. The highest scoring network had a total of 25 focus molecules and a score of 16 and this network is associated with energy production, nucleic acid metabolism and small molecule biochemistry; this top scoring network is shown in Fig. 5C where the canonical pathways that are associated with these data set and network were overlaid (and have a CP tag next to them).

### Adult crush vs old crush—what genes are differentially expressed in muscle of old mice following nerve crush in comparison with adult mice following nerve crush?

**Fig. 6:**
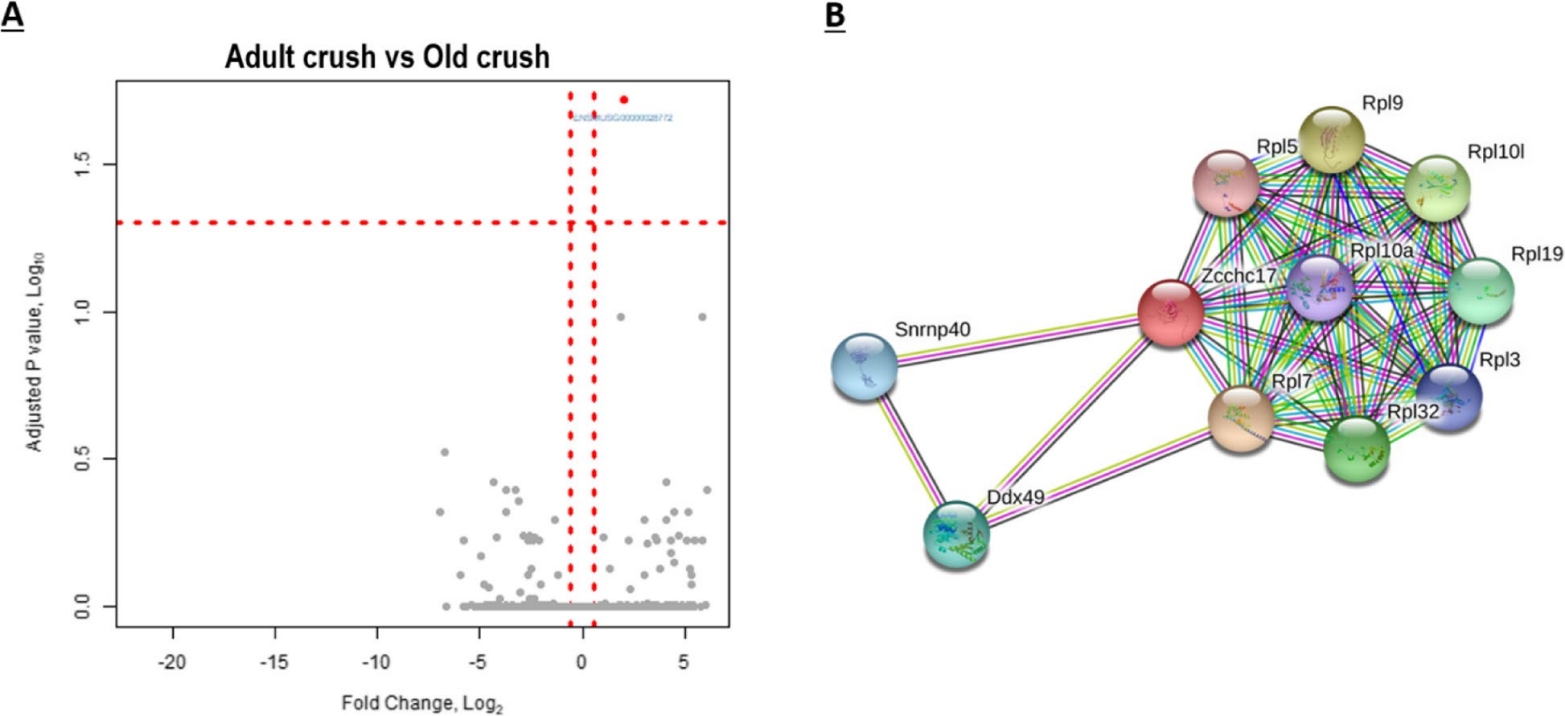
DEG between muscles of adult and old mice post-nerve crush revealed ZCCHC17 as the only DEG. **A** Volcano plot showing the -log10 FDR for difference in expression between adult crushed and old crushed skeletal muscle for each gene detected, plotted against the log2 fold-change. ZCCHC17 is the one gene with an FDR < 0.05 and depicted in red with associated ENSBMBL ID outlined. **B** Interaction network identifying the most commonly associated protein to protein interactions of the ZCCHC17 gene produced using the STRING functional enrichment analysis database

To directly explore how ageing might affect the muscle response to nerve injury, the DEG following crush from both adult and old cohorts were compared. 29,842 gene transcripts were analysed, but only one gene, Zinc Finger CCHC-Type Containing 17 (*ZCCHC17*, also termed *PNO40*), was differentially expressed (log2 fold-change of 1.99 ± 0.4, FDR < 0.05). This suggests a substantial harmonisation of gene expression in both cohorts at this time point following crush. This can be visualised in the volcano plot where by *ZCCHC17* is the single gene located at the top right of the plot in red (Fig. 6A). *ZCCHC17* was also shown by qPCR to be upregulated in muscle samples following nerve crush in old mice (data not shown).

IPA gene view categorises ZCCHC17 as a nucleolar protein involved in RNA and protein binding. The GO molecular functions that ZCCHC17 is associated with are metal ion binding, nucleic acid binding, protein binding and RNA binding. IPA proposes ZCCHC17 to be regulated by Kruppel Like Factor 3 *(KLF3*) and SET Nuclear Proto-Oncogene (*SET*), and binds to Syndecan Binding Protein 2 (*SDCBP2*), Zinc Finger And BTB Domain Containing 38 (*ZBTB38*), Jumonji Domain Containing 6 (*JMJD6*), amyloid precursor protein (*APP*), Pinin (*PNN*), Dynein Light Chain Tctex-Type 1 (*DYNLT1*), Nuclear Receptor Subfamily 2 Group C Member 2 (*NR2C2*), Cyclic GMP-AMP Synthase (*CGAS*), Autophagy Related 16 Like 1 (*ATG16L1*), HECT And RLD Domain Containing E3 Ubiquitin Protein Ligase 2 (*HERC2*), HECT Domain E3 Ubiquitin Protein Ligase 1 (*HECTD1*), Ribosomal RNA-processing protein 8 (*RRP8*), Ferritin Light Chain (*FTL*), Joining Chain Of Multimeric IgA And IgM (*JCHAIN*) and RNA-binding motif (*RBM*).

Expression data from the Human Protein Atlas show that *ZCCHC17* has a low tissue specificity, but the RNAseq databases suggest it is predominantly expressed in neuronal tissues. Phenotypes, diseases and traits associated with this gene include abnormal cell differentiation, early cellular replicative senescence and oxidative stress [83].

Protein-protein interactions of *ZCCHC17* were identified with STRING and the interacting partners are shown in Fig. 6B [50]. GO analysis of the ZCCHC17 1st order protein interactome revealed 14 significantly enriched biological process; see Table 6.

**Table 6:** Significantly enriched Biological GO terms associated with Zcchc17

Biological terms enriched in STRING Network	GO ID	FDR
Translation	GO:0006412	6.30E−11
Peptide metabolic process	GO:0006518	2.29E−10
Cytoplasmic translation	GO:0002181	2.46E−09
Ribosomal large subunit biogenesis	GO:0042273	4.82E−09
Ribosomal large subunit assembly	GO:0000027	9.76E−06
Gene expression	GO:0010467	1.26E−05
Cellular nitrogen compound metabolic process	GO:0034641	1.26E−05
Macromolecule metabolic process	GO:0043170	0.0003
rRNA processing	GO:0006364	0.00072
Maturation of LSU-rRNA	GO:0000470	0.00075
Liver regeneration	GO:0097421	0.00094
Primary metabolic process	GO:0044238	0.00097
RNA processing	GO:0006396	0.0019
RNA metabolic process	GO:0016070	0.032

### Adult crush vs old control—does nerve crush in adult mice replicate the gene expression changes seen with ageing in muscle from old mice?

The extensive motor neuron loss and NMJ degeneration seen in old mice associated with muscle loss prompted a comparison of the DEG found in AT muscles from control old mice with those seen post-nerve crush in adult mice. In order to investigate how similar the DEG changes that occur in muscle with ageing due to the presence of denervated muscle fibres in old age to those seen in muscle from adult mice following nerve crush, we compared these data and used statistical equivalence testing to obtain further information on the commonality between the two data sets.

**Fig. 7:**
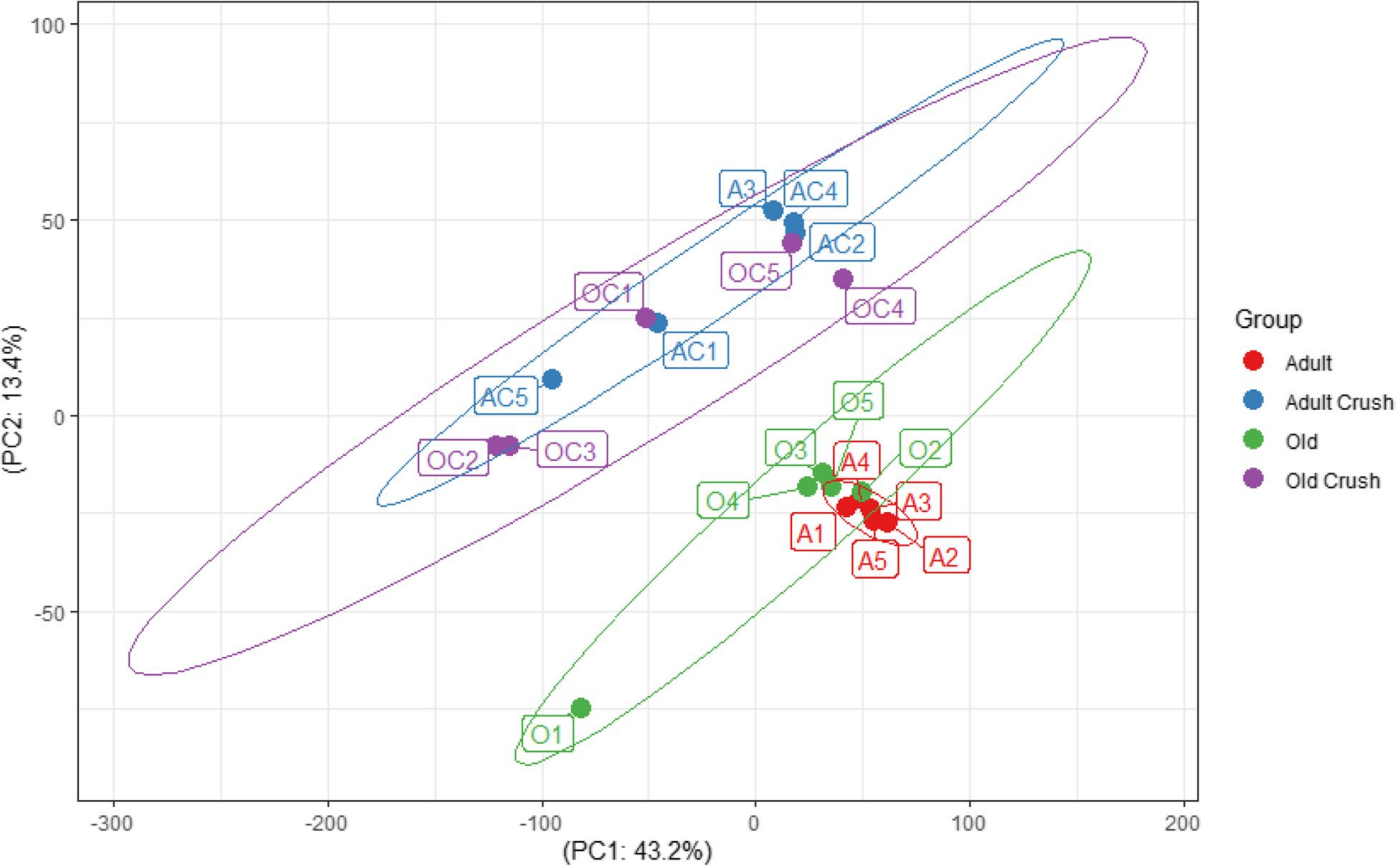
Principal component (PC) analysis of RNA sequencing data. Samples are defined by A for Adult, AC for Adult crush, O for Old and OC for Old crush with 5 samples for group

Principal component analysis (PCA) across all 4 datasets (adult, adult crush, old and old crush highlight variability between populations but do not inform on a gene by gene basis (Fig. 7)). The PCA performed on normalised count data shows a strong clustering of samples by treatment (crush vs control), but is not clearly separated by age. The adult crush samples (labelled AC1–5) and the old control samples (O1–5) do not overlap or appear to closely correlate. This graph shows a visual representation of the data based on normalised counts rather than a full DE analysis. Euclidean distance was calculated as a measure of cluster separation between the groups. The analysis indicated that the data from nerve crushed adult muscle were not significantly different from old control population (Table 7; *p* = 0.78).

**Table 7:** Cluster similarity using Euclidean distances was examined for all contrasts

	PCA	
	Euclidean distance	*p* value
Adult control vs adult crush	245.064	0.09
Adult control vs old control	218.38	0.04
Old control vs old crush	283.992	0.37
Adult crush vs old crush	281.844	0.36
Adult crush vs old control	240.868	0.78

**Fig. 8:**
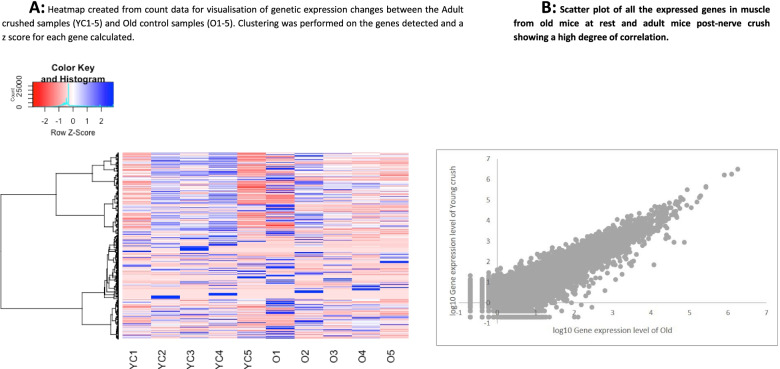
Heatmap and scatter plot of RNAseq expression analysis from adult crushed vs old control AT muscle samples. **A** Heatmap created from count data for visualisation of genetic expression changes between the 5 Adult crushed samples (YC1–5) and Old control (O1–5). Clustering was performed on the genes detected and a *z* score for each gene calculated and plotted. **B** Scatter plot of all the expressed genes in muscle from old mice at rest and adult mice post-nerve crush showing a high degree of correlation

Hierarchical clustering by genes (rows) and population (columns) shows genes acting in a similar manner (Fig. 8A). The mean count data for both groups is plotted as a scatter plot in Fig. 8B outlining how well the genes are correlated between the two groups with very few obvious outliers.

Equivalence testing was conducted to further explore the potential similarity between the two populations; this examined all the genes detected to assess what number of genes (and what genes) are statistically similar in expression between the adult crush vs old control populations. This non-parametric test used normalised gene counts and identified 2672/21,269 genes that were statistically similar in expression between the adult crush and the old control samples.

## Discussion

The aim of this study was to use RNAseq analysis to examine differences in gene expression underlying age-related decline in skeletal muscle in mice, to identify whether the differences seen in old mice were related to the presence of actively degenerating or regenerating nerves, and to identify differences between the acute transcriptional responses of muscle between old and adult mice subject to nerve crush that might indicate why muscle from old mice shows poor re-innervation following nerve damage. The post-crush samples were obtained at 3 days following peroneal nerve crush, a time point previously shown by us as associated with significant loss of the distal axonal structure and an increase in generation of peroxides by the AT muscle mitochondria, although AT mass is preserved at that stage [99].

The data obtained have identified DEG that occur in muscle with ageing, and show that all of the DEG found in muscle from old mice can be induced in adult mice by nerve crush. Only one DEG was found in adult mice following nerve crush compared with those seen in muscle of old mice following nerve crush indicating that differences between muscles from old and adult mice were minimised in the responses to crush injury.

This study revealed the DEG for each contrast, few of which have previously been explored with respect to skeletal muscle ageing and denervation but provided some interesting predicted pathway enrichments and potential upstream regulators and network analyses. Whilst a range of DEG were detected which could be controlling how adult and aged skeletal muscles respond to injury/denervation, it is important to appreciate the limitations of this approach. It cannot be presumed that all the DEG are from the myofibres since the muscle tissue analysed contain a number of other cells including neuronal tissue satellite cells, fibroblasts, terminal Schwann cells (tSCs), adipocytes and immune cells which, although present in low amounts, might contribute to the data obtained and to overcome this one could conduct single cell RNAseq, or possibly single nucleus RNAseq would be appropriate. It is important to realise that aged motor neurons have altered properties which could participate in the observed phenotype. It is known that during ageing, the number of satellite cells decline in mice [12, 22] in a fibre type specific manner; there are also increases in fibrous connective tissue and adipose tissue with age [7, 37]. While RNAseq is very powerful in identifying important genes and pathways, in common with other bioinformatic analyses, several databases/platforms were utilised with different strengths and weaknesses and have revealed potential therapeutic targets and pathways for further intervention. When exploring/comparing those GO or KEGG pathways to enriched IPA outputs, many are not exact overlaps or seen across all databases. This is due to the fact that many use databases with different inputs. Similarly, this study reports the changes at a transcriptional level, and it is recognised that this may not reflect those changes seen at protein level.

### Effect of age on DEG in murine skeletal muscle

The response of skeletal muscle to ageing was examined by comparison of DEG between muscles of adult and old mice. This comparison revealed 334 DEG, from a total of 29,842 genes identified. Only a small proportion of these genes have previously been studied in relation to skeletal muscle biology. Pathway enrichment analysis was undertaken to identify how such differential gene expression could contribute to age-related loss of murine skeletal muscle mass and function.

Several of the processes identified are linked to neurological degeneration or regeneration. Thus, the 5 most upregulated DEG found from the comparison between data from control muscle of adult and old mice were associated with different biological processes. Leucine-rich repeat kinase 2/PARK8 (*Lrrk2*) a gene associated to the neurological disorder Parkinson’s disease was found to be differentially expressed [33]. This gene has not been studied in detail with respect to skeletal muscle physiology but it is widely expressed and believed to have a key role in controlling neurite morphology and complexity as well as inflammation, protein degradation and maintaining mitochondrial homeostasis [16, 32, 110], all of which are known to be perturbed in skeletal muscle during ageing.

Another strongly upregulated DEG was ATPase Na+/K+ Transporting Subunit Alpha 4 (*Atp1a4*); this gene encodes an integral membrane protein responsible for establishing the electrochemical gradient of Na^+^ and K^+^ ions [1]. Gene-targeting studies on alpha subtype KO mice to the Na,K-ATPase pump have suggested they play a role in regulating Ca(^2+^) transients involved in muscle contraction [66], and this could suggest that this Atp1a4 DEG may contribute to impairments in SR Ca^2+^ release, impairments that have been suggested to explain deficits in physical performance in muscle from older animals [82].

Syntaxins, a family of SNARE proteins, are membrane proteins primarily localised to the presynaptic active zone and involved exocytosis of vesicles. Syntaxin 11 (*STX11*) was downregulated with age. Changes in syntaxins have been linked to impaired neurotransmission [35, 45, 96], but no previous work has associated syntaxin 11 with skeletal muscle ageing.

Other DEG identified were associated with processes such as DNA damage regulation (such as *Wdhd1*), but this has not previously been associated with muscle physiology [34]. The *Pros1* gene (Protein S) was found to be significantly downregulated in muscle of old mice. Little previous work has been reported on *Pros1*, but a study by Murton et al. [78] indicated this gene to be part of an enriched pathway observed in human skeletal muscle following concentric resistance exercise training.

KEGG analysis revealed both glutathione metabolism and AMPK signalling pathways as top enriched pathways. This was unsurprising since both pathways are acknowledged to play key roles in muscle homeostasis and loss in ageing [54, 75, 86]. AMPK signalling was significantly enriched using KEGG and IPA when assessing those DEG between adult and old skeletal muscle.

IPA upstream analysis identified interleukin-9 (*IL-9*) signalling as one of the top canonical pathways associated with ageing skeletal muscle. IL-9 and its receptor (IL-9R) are mainly expressed in neuronal and immune cells [55] and are activators of JAK/STAT signal transduction pathways linking cytokines to several biological processes. IL-9 is chiefly known to target immune cells and functions as a growth factor with anti-apoptotic activities [4] and this observation suggests that modulation of IL-9 may influence the motor nerve degeneration observed in skeletal muscle with age. IL-9 which is produced primarily by Th2 helper T cells is a prototypical cytokine associated with inflammation, so this may be due to immune cell activation within the skeletal muscle of old mice although this was not examined in detail. T cell infiltration into damaged skeletal muscle occurs rapidly following injury and a report by Cheng et al. [18] found that this infiltration begins at about 3 days following injury and remains until at least day 10 of regeneration so it is likely that the IL-9 here is a consequence of immune cell activity. Insulin receptor signalling was also the top enriched canonical pathway from the comparison between adult and old skeletal muscle. This is fully compatible with the well-known change in insulin resistance of muscle with ageing [39].

Several upstream regulators (UR) were also revealed using the advanced analytics in IPA. The UR identification methods examine links to DEG through coordinated expressions to potential UR that can be transcription factors or any gene or small molecule that has been observed experimentally to affect gene expression. The top UR detected between samples from old and adult mice was the key structural protein dystrophin (*DMD*). It appears surprising that this large protein was indicated in this type of analysis but UR analytics showed it to be associated with 4 target genes in the dataset (*COL1A2*, *H19*, *HSPA8 and PVALB*). Dystrophin appears to have regulatory roles in addition to the well-known structural role. This has been shown by studies in dystrophin-deficient (mdx) mice and many studies have now identified compensatory proteins to *DMD* which are thought to be involved in the maintenance of the neuromuscular junction architecture and in muscle homeostasis (Rouger et al. [89]; Hughes et al. [47]. Dumont et al. [26] also found that dystrophin has an essential role in the regulation of satellite cell polarity and asymmetric division. This is crucial for muscle regeneration and in the absence of dystrophin, myofibres become fragile and there is impaired regeneration due to intrinsic satellite cell dysfunction [13].

A study by Pannérec et al. [81] assessed gene expression in gastrocnemius muscles from aged rats and identified several “neuromuscular junction related” genes that were differentially expressed when comparing adult (8 months), early sarcopenic (18–20 months) and sarcopenic (22–24 months) muscle samples. Dystrophin *(DMD)* (as mentioned above) and *Thbs4* were deemed neuromuscular junction related. Thromonospondin (*Thbs4*), a protein that regulates skeletal muscle integrity and its susceptibility to muscular dystrophy [106], was significantly DE between adult and old muscle (log2FC of 1.27), with the same pattern of expression observed in the Pannérec et al. [81] dataset. Similarly when comparing the GO enriched terms from adult vs old DEG and that of Pannérec et al. [81], the common enriched biological processes included neuromuscular function, extra-cellular matrix remodelling and fibrosis, mitochondrial function and inflammation.

One of the top master regulators identified was *TARDBP*; this gene encodes TDP-43, a highly conserved and ubiquitously expressed protein involved in transcription and RNA splicing regulation. Hyper-phosphorylated and ubiquitinated TDP-43 deposits are associated with several neurodegenerative diseases where the deposits produced act as inclusion bodies in the brain and spinal cord [87]. Olivé et al. [79] have indicated that TDP-43 not only accumulates in myofibrillar myopathies, but plays a key role in altered microRNA processing in the abnormal protein production, modification and accumulation in protein aggregate myopathies and these data suggest that similarities could be occurring due to age. CCAAT enhancer binding protein beta (*CEBPB*) was also found to be a master regulator and this particular gene highlights the potential of satellite cell involvement with skeletal muscle ageing. Recent studies show *CEBPB* as a novel regulator of satellite cell self-renewal during muscle cell regeneration [58, 69]. The *CEBPB protein* has also recently been associated with ATF4 whereby it is thought to mediate muscle atrophy [27].

The pathways and specific DEG outlined above were obtained from examining muscle tissue and so a limitation of this study was that we were unable to confirm the cellular source and data on any changes in skeletal muscle fibre type proportions were not available in our current study. Murgia et al. [77] conducted a single fibre proteomic analysis of human fast and slow muscle fibre types and similarities between their results and ours highlighted the role of several pathways as well as individual proteins. Murgia et al. [77] emphasised the features of mitochondrial ageing in both slow and fast fibres, the role of stress response proteins as well those associated to proteostasis and the role of chaperones. Our data also confirm differences in several Glutathione Peroxidase genes (including *GPX3*) and Glutathione S-transferases genes (including *GTSP1*) as well as stress response genes (*ATP6V*) and several chaperones specifically heat shock proteins (including *HSPA8*) in a similar manner to the datasets of Murgia et al. [77], supporting the use of this approach.

### Effect of nerve crush on DEG in muscle from adult mice

7133 skeletal muscle genes showed differential changes in expression following crush injury to the peroneal nerve in adult mice. Experimental data has shown that sectioning of motor nerves or nerve crush leads to the rapid activation of degenerative pathways in the denervated muscle, including an increased mitochondrial generation of reactive oxygen species [76], increased generation of pro-inflammatory cytokines [46] and disruption of protein homeostasis [102]. The time course of on-set of these changes following nerve section indicates that they must occur even during the relatively short periods of denervation and subsequent re-innervation of muscle fibres that occur in younger animals. Thus, we have shown that the structure of the peripheral axons and NMJ are lost within 3 days of nerve sectioning; Staunton et al. [99] and Jang et al. [49] reported a remarkably large increase in muscle mitochondrial H_2_O_2_ generation following denervation and this increased mitochondrial H_2_O_2_ release is already apparent within 3 days of nerve transection. The effect of this activation of specific degradatory pathways is unclear although we speculate that initially this may reflect an attempt to restore innervation, since products such as cytokines are released from the muscle fibre and some cytokines have been proposed to stimulate axonal sprouting [28]. The large number of DEG found (approximately 24% of the total genes detected) reflects the major reconfiguration of the muscle induced by this procedure; changes include motor nerve degeneration, loss of the pre-synaptic NMJ, early phases of nerve regeneration and muscle atrophy. Amongst the DEG detected are key genes associated with all of these processes, including neuronal acetylcholine receptor subunit alpha-9 (*CHRNA9*), cholinergic receptor nicotinic gamma (*CHRNG*) and growth differentiation factor 5 (*GDF5*) and the canonical pathways identified include mTOR signalling which is key to regulation of muscle protein homeostasis. The KEGG analysis identified pathways induced in the muscle of adult mice following nerve crush that are associated with neurodegenerative disorders, such as Huntington’s, Parkinson’s and Alzheimer’s diseases (Fig. 4B). While this may suggest common neurodegenerative pathways occur in these disorders and sarcopenia, it may simply reflect the on-going basic neurodegeneration and regeneration in these disorders which is recapitulated by experimental nerve crush. Motor unit reorganisation and inflammatory changes in motor neurons are known to decrease conduction velocity and amplitude of compound muscle action potential leading to neuromuscular impairment and this could be why such neurological diseases have been identified here [56]. It was then not surprising that IPA outputs revealed upstream regulators and master regulators that too have been strongly implicated in neurodegenerative disease such as PPARG coactivator 1 alpha (*PPARGC1A*) [21, 51, 73] and the mitochondrial uncoupling protein Thermogenin (*UCP1*) [19, 44, 53].

Most surprisingly, a comparison of the DEG in skeletal muscle resulting from nerve crush in adult mice with the DEG in skeletal muscle of control old compared with control adult mice revealed that all of the 344 DEG associated with ageing in control muscle were induced by nerve crush in muscles of adult mice although the full extent of DEGs seen in adult mice with crush is considerably greater. Thus, the DEG seen in control muscles of old mice do not reflect the full extent of gene changes following crush in adult mice. These data indicate that, in terms of DEG, all of the changes seen in muscle during ageing are reproduced as part of the pattern of DEG seen in adult mice by crush of the peroneal nerve.

These results emphasise the critical importance of motor nerve loss and regeneration (i.e. motor unit turnover) in the transcriptional changes seen with ageing in skeletal muscle and fundamentally influence the way in which ageing induced changes in skeletal muscle gene expression are interpreted. This conclusion about the importance of motor neuron and motor unit loss in the causes of ageing related muscle loss has been implied previously on the basis of physiological [10] and structural changes [5, 108], but has not previously been highlighted by analysis of DEG.

### Does ageing affect the way in which skeletal muscle responds to nerve crush injury?

Six hundred ninety-nine DEG were detected in muscle from old mice following nerve crush. This was much less than the 7133 DEG found in muscle of adult mice following nerve crush despite the similar levels of total genes detected (29,842 and 29,844 in muscles of adult and old mice respectively). The implication of this is that muscle from old mice had a diminished response to nerve crush. Muscle from aged mice has been claimed to have multiple interacting dysfunctional systems [72], such as a change in proteostasis [31], infiltration of fat tissue and connective tissue into skeletal muscle [104], mitochondrial dysfunction [48], inability to evoke a cytoprotective response [107], reduced number of satellite cells [12, 95], increased ROS production [80] and increased inflammation [29], which could all contribute to this diminished response mechanism.

In contrast, when the muscles from adult and old mice post nerve crush were directly compared there was only one DEG (ZCCHC17) which was upregulated in the muscle from old mice (Log2 Fold change 1.99, BH-adjusted *p* value 0.01). This Zinc Finger CCHC-Type Containing 17 (*ZCCHC17*) protein (also known as *PNO40*) is localised to the nucleolus and interacts with multiple metal ions, nucleic acids, proteins and RNA binding proteins [14]. *ZCCHC17* has recently been proposed as a master regulator in Alzheimer’s disease and is expressed in neuronal cells where levels are seen to drastically decrease early in AD, before significant neuronal cell loss or gliosis. ZCCHC17 is also believed to support the expression of a network of synaptic genes and linked to calcium signalling and protein mannosylation [64, 103]. This study is the first to identify, on the basis of DEG, a common link between this ageing-related disorder and sarcopenia and suggests *ZCCHC17* may be a potential driver for both of these disorders.

An original aim of the study was to identify differential responses between skeletal muscle of old and adult mice post nerve crush that might explain the age-related differences in nerve repair capacity. It was hypothesised that a contributor to the defective re-innervation that occurs in muscle from old mice is a failure to release appropriate “sprouting factors” from muscle. Identification of specific axonal “sprouting factors” released from transiently denervated muscle fibres (and which are not appropriately released from muscle of old mice) is thought to be potential therapeutic targets to help retain appropriate innervation of muscle fibres during ageing. It was anticipated that neurotrophins such as NGF, BDNF, NT3 and NT5 would be identified. Whilst several typical neurotrophins were detected within the muscle bulk of adult and old mice and could be acting as potential regulators of the maintenance, function and regeneration of skeletal muscle, they were not differentially expressed. Other genes that were detected that could also be playing a role in NMJ maintenance and repair included Neurofascin (*Nfasc*)—a cell adhesion protein that is involved in synapse formation, axonal guidance and neuronal-glial cell interactions [15, 20]; Neuroplastin (*NPTN*—a key neuronal cell adhesion molecule associated with neurite outgrowth and neuronal and synaptic plasticity [3]; and Neuroglobin (*NGB)* which is believed to be a stress sensor and promotes neuronal survival [2, 88].

Although we only identified one DEG (ZCCHC17) by direct comparison of DEGs in muscles from old mice following nerve crush to muscles of adult mice following nerve crush, there were 7133 DEG induced by nerve crush in muscles of adult mice compared with 699 DEG induced by nerve crush in muscles from old mice. The identification of only a single DEG in the direct comparison of nerve crushed old vs nerve crushed adult muscle is therefore surprising and presumably reflects the substantial increased variability in baseline gene expression that occurs with ageing.

A comparison of the top 10 canonical pathways identified by IPA in muscles from old nerve-crushed vs control old mice (Table 5) with those from adult nerve-crushed vs control adult mice (Table 4) revealed only one pathway in common (sirtuin signalling) and it therefore seems likely that further detailed analysis of the DEG that are induced by nerve crush in the adult vs those induced by nerve crush in the old may indicate potential gene contributors to the diminished DEG response in muscle from old mice in responses to nerve crush, and hence possible muscle contributors to diminished nerve regenerative capacity.

The findings of this study should be considered in light of some limitations. The availability of mice only allowed the examination of these effects in male mice. The examination of these effects in female mice is warranted although there is little evidence of any effect of sex on the rate of denervation seen in old age or differential effects following acute denervation. A lack of tissue availability excluded analysis of the effect of a change in muscle fibre type composition on our findings although the AT is predominantly type II and so we anticipate that fibre type will have played little role in our findings. The study focussed on the responses to nerve crush at a relatively early time point and so we were not able to exclude any additional effects at later time points. Data from the analysis of whole muscle tissue should always be examined with caution since muscle tissue contains a range of cell types which contribute to the local environment. An added complexity of analysis of RNASeq data is that in some instances this does not reflect the protein content of the tissue. However, the data are important in identifying differential control of critical pathways and provide a valid outcome despite the caution needed in interpretation.

In conclusion, this study of differentially expressed genes in AT muscles from adult and old mice and adult and old mice that had undergone a crush injury to the peroneal nerve has characterised the transcriptional changes underlying age-related decline in skeletal muscle and differences between the responses of muscle from adult and older mice to nerve crush. Data have identified 344 DEG that occur in muscle with ageing and indicate that all of these DEG can be induced in muscle from adult mice by nerve crush suggesting a major component of nerve degeneration/remodelling in the changes in muscles of old mice. Although only one DEG was found by direct comparison of muscles from old mice following nerve crush and muscles of adult mice following nerve crush, there were 7133 DEG induced by nerve crush in muscles of adult mice compared with 699 DEG induced by nerve crush in muscles from old mice and further analysis of these genes may indicate key differences in the DEG response of skeletal muscle from old mice which underlie the diminished ability of muscle from old mice to repair following nerve injury.

## Supplementary Information

**Additional file 1.** Supplementary Table S1

## Data Availability

Full data are available on the EBI array express database with accession number: E-MTAB-10601.
