# Supplementary material for: Skeletal muscle transcriptomics identifies common pathways in nerve crush injury and ageing
Source: Skelet Muscle. 2022 Jan 29;12:3. doi: 10.1186/s13395-021-00283-4 (PMC8800362; doi:10.1186/s13395-021-00283-4)
Supplement: Supplementary file 1 — Additional file 1. Supplementary Table S1 [file 13395_2021_283_MOESM1_ESM.pdf]

Supplementary table 1: Common DEG between Adult and Old mice using the SarcoAtlas platform. The 28 genes listed below are common between the adult (6month) and old (26month old) data produced from this study and from the SarcoAtlas - 'Aging-CR-RM' control mice data base (GEO accession number: GSE139204).

Ensembl gene ID	Gene name	FDR	log2 Fold Change
ENSMUSG00000036046	5031439G07Rik	0.026603381	0.748101918
ENSMUSG00000074264	Amy1	0.037774657	-1.307878632
ENSMUSG00000041460	Cacna2d4	0.024393981	1.686974117
ENSMUSG00000020722	Cacng1	0.005390966	-1.213623985
ENSMUSG00000048701	Ccdc6	0.044572598	0.891253522
ENSMUSG00000039084	Chad	0.010575832	3.311390977
ENSMUSG00000029661	Col1a2	0.024393981	1.802937022
ENSMUSG00000026837	Col5a1	0.028210175	1.595960771
ENSMUSG00000025366	Esyt1	0.005446908	0.893483419
ENSMUSG00000022200	Golph3	0.043901614	1.129390081
ENSMUSG00000027346	Gpcpd1	0.001887492	1.183694444
ENSMUSG00000015656	Hspa8	0.024954623	1.228141897
ENSMUSG00000024210	Ip6k3	0.024393981	1.271108083
ENSMUSG00000031740	Mmp2	0.042441263	1.79621396
ENSMUSG00000019933	Mrln	0.036039132	-1.103714284
ENSMUSG00000014776	Nol3	0.025944996	-1.323974165
ENSMUSG00000019916	P4ha1	0.001634674	1.414809161
ENSMUSG00000021143	Pacs2	0.042534808	1.016588496
ENSMUSG00000021140	Pcnx	0.035423763	1.41187465
ENSMUSG00000028273	Pdlim5	0.033718065	1.05752822
ENSMUSG00000024145	Pigf	0.021173193	2.107970385
ENSMUSG00000022216	Psme1	0.024393981	-0.809860243
ENSMUSG00000020460	Rps27a	0.024954623	-0.963295973
ENSMUSG00000022462	Slc38a2	0.039451538	1.565301937
ENSMUSG00000063919	Srrm4	0.0000286	-2.235730788
ENSMUSG00000021702	Thbs4	0.018458307	1.269035079
ENSMUSG00000032501	Trib1	0.025742679	1.21045814
ENSMUSG00000036769	Wdr44	0.036941415	1.90061141
